# Pulse Oximeter App Privacy Policies During COVID-19: Scoping Assessment

**DOI:** 10.2196/30361

**Published:** 2022-01-27

**Authors:** Rachele Hendricks-Sturrup

**Affiliations:** 1 Future of Privacy Forum Washington, DC United States; 2 Duke-Margolis Center for Health Policy Washington, DC United States

**Keywords:** COVID-19, pulse oximeters, mobile apps, mHealth, privacy

## Abstract

**Background:**

Pulse oximeter apps became of interest to consumers during the COVID-19 pandemic, particularly when traditional over-the-counter pulse oximeter devices were in short supply. Yet, no study to date has examined or scoped the state of privacy policies and notices for the top-rated and most downloaded pulse oximeter apps during COVID-19.

**Objective:**

The aim of this study was to examine, through a high-level qualitative assessment, the state and nature of privacy policies for the downloaded and top-rated pulse oximeter apps during the COVID-19 pandemic to (1) compare findings against comparable research involving other mobile health (mHealth) apps and (2) begin discussions on opportunities for future research or investigation.

**Methods:**

During August-October 2020, privacy policies were reviewed for pulse oximeter apps that had either at least 500 downloads (Google Play Store apps only) or a three out of five-star rating (Apple Store apps only). In addition to determining if the apps had an accessible privacy policy, other key privacy policy–related details that were extracted included, but were not limited to, app developer location (country); whether the app was free or required paid use/subscription; whether an ads disclosure was provided on the app’s site; the scope of personal data collected; proportionality, fundamental rights, and data protection and privacy issues; and privacy safeguards.

**Results:**

Six pulse oximeter apps met the inclusion criteria and only 33% (n=2) of the six apps had an accessible privacy policy that was specific to the pulse oximeter app feature (vs the app developer’s website or at all). Variation was found in both the regulatory nature and data privacy protections offered by pulse oximeter apps, with notable privacy protection limitations and gaps, although each app provided at least some information about the scope of personal data collected upon installing the app.

**Conclusions:**

Pulse oximeter app developers should invest in offering stronger privacy protections for their app users, and should provide more accessible and transparent privacy policies. This is a necessary first step to ensure that the data privacy of mHealth consumers is not exploited during public health emergency situations such as the COVID-19 pandemic, where over-the-counter personal health monitoring devices could be in short supply and patients and consumers may, as a result, turn to mHealth apps to fill such supply gaps. Future research considerations and recommendations are also suggested for mHealth technology and privacy researchers who are interested in examining privacy implications associated with the use of pulse oximeter apps during and after the COVID-19 pandemic.

## Introduction

Symptom and health behavior tracking applications or smartphone apps continue to grow in popularity along with government interest and oversight over the privacy practices of such apps [1]. Notably, recent research has raised concerns about the privacy and security of information provided and exchanged via mobile health (mHealth) and wellness apps in general, especially apps that target certain disease or patient populations. For instance, a recent study that examined 29 commercial smartphone apps developed for individuals coping with migraines or headaches (diary and relaxation apps) concluded that the apps shared information with third parties, while also noting that there are few legal protections that protect against the sale or disclosure of app user information to third parties [2]. Another study deployed a semiautomatic app search module to examine the privacy-related information of diabetes-focused apps available via Android, discovering that nearly 60% of the 497 apps surveyed requested permissions that significantly risk user data privacy and that 28.4% of the apps did not house their privacy policies on a website [3]. Several other recent studies discovered similar variation in findings for a variety of broadly available mHealth apps, which are discussed further below [4-15].

Pulse oximeter apps became of interest to consumers during the COVID-19 pandemic, particularly when traditional over-the-counter pulse oximeter devices were in short supply, as consumers sought to personally monitor themselves for hallmark symptoms of SARS-CoV-2 infection (eg, low blood oxygen saturation) [16,17]. Traditional medical-grade pulse oximeters function using a clamp that can be placed over a person’s fingertip, which then shines a light over the fingertip to measure blood oxygen saturation. Some pulse oximeter apps connect with traditional, medical-grade pulse oximeter devices via Bluetooth or USB and can export data/records to other devices. The US Food and Drug Administration (FDA) defines a pulse oximeter as “a device used to transmit radiation at a known wavelength(s) through blood and to measure the blood oxygen saturation based on the amount of reflected or scattered radiation” [18]. To obtain a pulse oximetry reading, pulse oximeter devices project a light at a specific wavelength that is shined over a specific area of a person’s body while the device measures how much light is absorbed (vs transmitted) by the blood cells within that area of the body. This process is somewhat similar to how mobile apps collect these same measurements, and studies have examined the differences in performance between pulse oximeter mobile apps and medical/hospital-grade pulse oximeters [19,20].

Consumer Reports recently outlined the pros and cons of using pulse oximeter apps, noting a specific app that is available on smart Android phones, called the Pulse Oximeter-Heart Rate Oxygen Monitor App, developed by digiDoc Technologies. This app is meant to be used only for athletic or fitness purposes and not for medical purposes given its technical performance limitations [21]. However, pulse oximeter apps that rely on flash and camera lighting to measure blood oxygen saturation are not always reviewed and approved by regulatory authorities such as the US FDA. Pulse oximeters recently underwent increased scrutiny during the COVID-19 pandemic due to research highlighting racial bias in pulse oximeter devices developed and trained on nonracially diverse populations of individuals, thereby prompting the need for further investigation regarding the scientific validity and accuracy of pulse oximeters [22].

Traditional, over-the-counter pulse oximeters became in short supply during the pandemic amid supply chain shortages. Yet, no study has been published to date broadly examining the privacy policies of pulse oximeter apps at the height of the broad societal impact of the COVID-19 pandemic (mainly, during 2020). Specifically, the literature offers no high-level qualitative assessment on the state or nature of privacy policies for the most downloaded and top-rated pulse oximeter apps during this challenging period. Therefore, the aim of this study was to address this gap to compare findings against comparable research involving other mHealth apps, which can begin discussions on how future research can fill important knowledge gaps about the state of privacy practices for pulse oximeter apps during and after the COVID-19 pandemic.

## Methods

In August 2020, the Google Play Store and Apple Store were searched to scope and identify pulse oximeter apps that had either at least 500 downloads (Google Play Store apps only) or a three out of five-star rating (Apple Store apps only). The total number of pulse oximeter apps available on both the Google Play Store and Apple Store was not tallied for purposes of the analysis. Under the direction of the author, two junior analysts reviewed privacy policies for pulse oximetry–specific apps that met the inclusion criteria between August and October 2020.

The following information was extracted from policies and statements found on the app developers’ publicly available websites and respective app stores: software purpose; developer location (country); whether the app was free or required paid use/subscription; mobile device access permissions stated on the app’s download site; whether an ads disclosure was provided on the app’s site; scope of personal data collected; how personal data are collected; who can access personal data; why personal data are used; where the data are stored; how long the data are stored; proportionality, fundamental rights, and data protection and privacy issues; privacy safeguards; and whether the privacy policy was accessible via the app store.

This specific information was extracted to align with our prior work to examine the extent to which each pulse oximeter app “appropriately and ethically balanced public health and safety with privacy risks and other interferences with civil liberties” during the COVID-19 pandemic [23].

These details were captured and summarized independently by the same two junior analysts and the summary was reviewed by RHS for accuracy and clarity. The finalized summary of findings was not reviewed and verified by the developers of the apps that met the inclusion criteria for further accuracy.

## Results

### Descriptive Assessment

Six apps in total met the study-specific inclusion criteria. Three of these six apps connect to or are compatible with an externally associated oximeter device. Among these three, only one provided a statement of FDA approval as a pulse oximeter device (EMAY Bluetooth Pulse Oximeter). The app developer’s headquarter locations were disclosed for all except one of the six apps (OxyCare-[Pulse Oximeter]); apps were developed in Vietnam, Spain, the United States, China, and Canada. Two apps required payment to either download or access certain features within the app (Pulse Oximeter-Beat & Oxygen and Oxxiom).

### Privacy Notice Assessment

Table 1 provides a full summary of privacy policy provisions and considerations for each of the six pulse oximeter apps reviewed.

Only two of the apps covered in this review (Pulse Oximeter-Beat & Oxygen and Kenek Edge) had privacy policies that were accessible directly via the app store. The other four apps reviewed (Oximeter, OxyCare [Pulse Oximeter], Oxxiom, and EMAY Bluetooth Pulse Oximeter) either did not have privacy policies that are accessible directly via the app store or did not have an accessible privacy policy that is specific to the pulse oximeter app. However, one app offered a user guide that contains user privacy guidance (Oxxiom). One app’s privacy policy is specific to the developer’s website versus the pulse oximeter app (EMAY Bluetooth Pulse Oximeter).

All six apps reviewed provided some information about the scope of personal data collected upon installing the app. All but one app (OxyCare [Pulse Oximeter]) specifically described *how* personal data are collected, *who* can access the personal data, *why* personal data are used, and *where and for how long* personal data are stored. Half of the apps reviewed (Pulse Oximeter-Beat & Oxygen, Oximeter, and Kenek Edge) provide an ads disclosure directly on the app download site. Two apps (OxyCare-[Pulse Oximeter] and Oxxiom) did not disclose deidentification commitments within the scope of proportionality, fundamental rights, and data protection and privacy issues. None of the apps’ policies explicitly stated if personal data would be used for research purposes. Only one app’s policy (Oximeter) explicitly stated that personal data are deleted once the app user permanently deletes the account. The five app developers that described *who* can access personal data in their privacy notices (excluding OxyCare [Pulse Oximeter]) discussed circumstances in which personal data are collected from and used by nonusers (ie, third-party service providers, advertising partners). Two of those five apps explicitly describe personal data access/use by law enforcement (Oximeter and Kenek Edge). Data collection and use for four of the apps are explicitly “opt-in” (Pulse Oximeter-Beat & Oxygen, Oximeter, Oxxiom, and Kenek Edge) and one app explicitly recommends disabling cookies as a privacy safeguard for personal data (EMAY Bluetooth Pulse Oximeter).

**Table 1:** Summary of pulse oximeter app privacy policy provisions reviewed during August-October 2020.

Category	Pulse Oximeter-Beat & Oxygen	Oximeter	OxyCare-(Pulse Oximeter)	Oxxiom	EMAY Bluetooth Pulse Oximeter	Kenek Edge
Software purpose	General digital health management app that helps users personally check their blood oxygen level and heart rate at any time	General digital health management app that helps users see the percentage of breathable oxygen at their current altitude and check what percentage of oxygen they are breathing	Digital health app that connects to traditional, medical-grade pulse oximeters via Bluetooth or USB	Digital health app that works only with the Oxxiom pulse oximetry system/device	Digital health app that allows users to transfer the pulse oximetry and heart rate data from the EMAY Bluetooth Pulse Oximeter device (Food and Drug Administration–approved) to smartphones	General digital health management app that helps users measure their blood oxygen and heart rate using a hospital-grade finger sensor that can be attached to users’ mobile phones or tablets
Developer location (country)	Vietnam	Spain	Not disclosed	United States	China	Canada
Free/Paid	Free to install but charges per feature offered within the app	Free to install and use	Free to install and use	Charge to install; pulse oximeter sold separately	Free to install and use	Free to install and use
Mobile device access permissions stated on app download site	Storage; Wi-Fi connection information; wearable sensors/activity data; photos, media, and files; receive data from internet; full network access; prevent device from sleeping; view network connections; run at startup; control vibration	Location; photos, media, and files; storage; view network connections; full network access	Location; photos, media, and files; storage; pair with Bluetooth devices; access Bluetooth settings	Users may post, upload, store, share, send, or display photos, images, video, data, text, comments, and other information and content (“Your Content”) to and via the app, which would grant the app a nonexclusive, transferable, sublicensable, worldwide, royalty-free license to use, copy, modify, publicly display, reproduce, translate, and distribute user content	Not disclosed	Location; weblogs; IP address; web browser information; date and time user accessed or left the developer’s website and which pages the user viewed; behavioral data (eg, sleep patterns); user communication records with the developer; personal information (eg, name, age, gender, height, and weight)
Ads disclosure on app download site?	Yes	Yes	No	No	No	Yes
Scope of personal data collected	“Registration” data (eg, name, email); “transaction” data (eg, purchases, offer responses, downloads); “help” data; app use (eg, heart rate, steps, flights climbed, age, height, weight); other data (eg, mobile device type, unique device ID, IP address, mobile operating system, mobile internet browsers)	“Account” data (eg, username, password, email); “additional” data (eg, biography, location, website, picture, address book); location data (eg, mobile or IP address); “log data” (eg, IP address, browser type, operating system, referring webpage, pages visited, location, mobile carrier, device information, search terms, cookies)^a^	Location (approximate via network and precise via GPS); USB storage (photos, media, files)^a^	Date and times of measurements; SpO2^b^, PR^c^, and PI^d^ measurements; sale information (eg, shipping address, contact information, credit card information)	Deidentified “basic” web server visitor information (eg, IP address, browser details, timestamps, referring pages)	Visit data (eg, location data, weblogs and other communication data, IP address, web browser information, date and time accessed); form data (eg, name, email); sleep data (eg, actions, behaviors, treatments, medication, and general wellness); identifying information (eg, email, device ID, site password); personal information (eg, name, age, gender, height, weight); location information
How personal data are collected	Via individuals (account creation or contacting the app); automatic app collection (eg, device, IP address); and third-party tracking technology (eg, cookies)	Via “various websites, email notifications, apps, buttons, widgets, ads, and commerce services”^a^	Not disclosed	Self-reported and self-uploaded	Tracking via cookies	Via individuals (account creation, contacting the app/site); automatic collection (eg, device, IP address); and third-party tracking technology (eg, cookies)
Who can access personal data	Authorized employees and contractors, service providers, app partners, advertisers, advertising networks. Users can opt-out from third-party use of data by uninstalling the app	If the user decides to publish the information, it will be public: service providers, third-party apps, and websites when the user links accounts, sellers of goods and services, law enforcement^a^	Not disclosed	Third-party payment service providers and authorized third-party e-commerce websites	Advertising partners and other third parties who use cookies	Access via business transfers, law enforcement, and via consent to third parties. Customer PII^e^ is not available to third-party advertisers; however, these third parties may automatically collect other information via cookies
Why personal data are used	To contact individuals, advertise relevant products and services, to use the app	To provide the app services while improving them over time and to provide relevant advertising^a^	Not disclosed	To provide app services	For routine administration and maintenance purposes	To contact individuals, advertise via third parties, perform the app’s services, and comply with the law
Where the data are stored	Internal memory of the user’s cellular device. Data processing takes place in the United States	Internal memory of the user’s device(s). Data processing takes place in the United States and any country where the app operates^a^	Not disclosed	Internal memory of the user’s iOS device	Not disclosed	Internal memory of the user’s devices; otherwise, not disclosed
How long the data are stored	Data for advertising purposes are stored as long as the app is installed on the mobile phone	If the user permanently deletes the account, then the data are deleted. Log data are deleted after a few months^a^	Not disclosed	Credit card information is not stored	Not disclosed	Not disclosed
Proportionality, fundamental rights, and data protection and privacy issues	Only aggregated, anonymous data are “periodically” transmitted to third parties. Advertisers will only have access to “Automatically Collected Information,” which is the device’s unique ID, IP address, mobile operating system, type of mobile browsers, and app use information	Nonprivate, aggregated, or “otherwise nonpersonal information” will be shared or disclosed^a^	Not disclosed	Not disclosed	User’s personal information cannot be used to identify specific visitors	Individuals can visit the app/website without revealing any personal information
Privacy safeguards	The app is opt-in. Physical, electronic, and procedural safeguards of data (eg, authorization process)	The app is opt-in^a^	Not disclosed	The app is opt-in	The app recommends disabling cookies	The app is opt-in. The developer has a “commercially suitable physical, electronic, and managerial procedure” to safeguard and secure collected information
Privacy policy accessible via app store? [24]	Yes [25]	No (same app developer’s Privacy Policy: RamLabs) [26]^a^	No	No [27]	Yes, although specific to the company website versus the app [28]	Yes [29]

^a^Information taken from the app developers’ general privacy policies; the policy could apply to the pulse oximeter app reviewed or a different app made by the developer.

^b^SpO2: oxygen saturation.

^c^PR: pulse rate.

^d^PI: perfusion index.

^e^PII: personal identifiable information.

## Discussion

### Principal Findings

The present findings fill an important literature gap regarding the privacy policies of pulse oximeter apps during the COVID-19 pandemic. These findings are largely consistent with trends observed in prior research that has examined the accessibility, structure, and substance of commercial mHealth apps’ privacy policies [2-15]. Namely, the top-rated or the most downloaded pulse oximeter apps during the COVID-19 pandemic either did not provide accessible privacy policies via the app store or did not provide privacy policies that were specific to the pulse oximeter app being offered. Thus, the present findings seemingly align with observations seen in recent assessments of privacy policies for a variety of mHealth apps. Although each pulse oximeter app provided some information to users about their scope of data collection, what is perhaps most concerning from a privacy standpoint is that all but one app (OxyCare [Pulse Oximeter]) provided privacy disclosures that are consistent with current privacy recommendations and best practices as well as policy-based guidance.

### Limitations

There are limitations to the present analysis and findings such that the observations reported herein are limited to only the highest rated or most downloaded pulse oximeter apps, which effectively excludes pulse oximeter apps that have lower ratings or are downloaded less frequently. In addition, this analysis did not include technical verification and quality assessment criteria for the apps, such as pulse oximeter app usability. Within these limitations are opportunities for further research to explore these important components as a critical next step to this broad analysis. This study was also cross-sectional in time such that it was intentionally limited to capture the state of pulse oximeter app privacy policies at the height of the COVID-19 pandemic when traditional, over-the-counter pulse oximeters were in short supply. Future research should examine if and the extent to which popular pulse oximeter app privacy policies have been either developed or updated.

### Alignment With Prior Research Examining the Privacy Policies of mHealth Apps

Several recent studies examined privacy policies and notices for a wide range of mHealth apps, noting trends that are similar to those found in the present analysis of pulse oximeter apps during COVID-19 [2-15]. The Future of Privacy Forum also published a similar study in a 2016 white paper, where they examined whether the most popular free and paid mHealth apps “provided users with access to a privacy policy, and whether the privacy policy was linked from the app’s listing page on the iOS [Apple] and Android app marketplaces” [30]. Therefore, the present analysis offers an opportunity to understand how the overall accessibility of privacy policies and notices for pulse oximeter apps during the COVID-19 pandemic compare with that of other health apps generally based on findings from comparable work published within the past 5 years (see Table 2).

**Table 2:** Comparison of present findings against comparable and prior privacy policy research focused on mobile health (mHealth) apps.

Study	mHealth app category surveyed	Apps meeting inclusion criteria, N	mHealth apps surveyed with an accessible mHealth app privacy policy, n (%)
This study	Pulse oximeter apps during COVID-19	6	2 (33)
FPF Mobile Apps Study [30]	Health and fitness, period tracking, sleep aid	25	19 (76)
Flors-Sidro et al [3]	Diabetes	497	139 (28.0)
O’Loughlin et al [5]	Depression	116	57 (49.1)
Grindrod et al [6]	Medication use and management	185	63 (34.1)
Rosenfeld et al [8]	Dementia	72	33 (46)
Huckvale et al [12]	Mental health (depression and smoking cessation)	36	25 (69)
Bachiri et al [15]	Pregnancy monitoring	38	18 (47)

The findings of this study showed a relatively low percentage of the most downloaded or top-rated pulse oximeter apps during COVID-19 that provided an accessible privacy policy (33%) compared with the average for the current trend seen in the literature for various mHealth apps (50%). This is problematic given that pulse oximeter apps grew in popularity during the COVID-19 pandemic, leaving pulse oximeter app users with an overall low degree of certainty about the privacy and security of their personal data that could be collected, shared, or processed by or via the apps.

### Future Opportunities and Priorities for Privacy Researchers and App Developers

Based on the present findings, it is recommended that future privacy research on pulse oximeter apps involve a deeper comparative analysis that would investigate the effectiveness of available privacy policies and/or offer a more technical analysis of privacy and security implications. Future work might also involve a systematic review, meta-analysis, or meta-synthesis of mHealth apps to more robustly capture and compare the state and substance of privacy policies and notices for mHealth apps, including pulse oximeter apps. Moreover, given that (1) certain pulse oximeter app user data could be considered as sensitive data under the EU General Data Protection Regulation (GDPR), and (2) each of these apps could function within the European Union and must therefore comply with the EU GDPR, future work should involve a robust risk assessment of pulse oximeter app and other mHealth app privacy policies against specific articles within the EU GDPR, most notably articles focused on user informed consent, data minimization, legal basis or grounds for data collection, data subjects’ rights, and consequential areas [31]. Lastly, pulse oximeter app developers should clarify within their privacy policies their purpose and need to collect sensitive information (eg, geolocation data, browsing data, address book data), as it may be unclear or not intuitive among users why the pulse oximeter app would need to collect such data to provide its intended services or experience to its users, and thus may be perceived as privacy-invasive.

### Conclusion

It is clear from the present review and related literature that mHealth apps, including pulse oximeter apps, hold vast opportunities—and perhaps necessity during and after the COVID-19 pandemic—to make their privacy policies more robust and aligned with these current privacy best practices and regulatory requirements. As the practice of medicine becomes increasingly digitized, offering consumers greater options to self-engage in health monitoring and data reporting using personal smartphones, the privacy and security of person-generated health data and traditional health become tantamount. Robust mHealth app consumer or user privacy protections, including, but not limited to, having an accessible and transparent privacy policy, are therefore needed to ensure that the data privacy of mHealth consumers cannot become exploited during public health emergency situations such as the COVID-19 pandemic, if patients and consumers feel compelled to purchase and download mHealth apps in response to short supplies of more traditional, over-the-counter personal health monitoring devices.

## Abbreviations

FDA: Food and Drug Administration
GDPR: General Data Protection Regulation
mHealth: mobile health
